# Comparison of the Blockbuster^TM^ and Air-Q^®^ Supraglottic Airway Devices as a Conduit to Blind Endotracheal Intubation in Paediatric Patients: A Randomised Control Trial

**DOI:** 10.4274/TJAR.2026.252084

**Published:** 2026-08-28

**Authors:** Arunima Pattanayak, Abhyuday Kumar, Chandni Sinha, Neeraj Kumar, Ajeet Kumar, Bindey Kumar, Rajnish Kumar

**Affiliations:** 1 All India Institute of Medical Sciences Patna, Department of Anaesthesiology, Patna, India; 2 India Gandhi Institute of Medical Sciences Patna, Department of Paediatric Surgery, Patna, India

**Keywords:** Airway management, fiberoptic, intubation, paediatric anaesthesia, supraglottic device

## Abstract

**Objective:**

Only two devices [air-Q® intubating laryngeal airway (ILA) and AmbuAura-i] have been studied previously for blind endotracheal intubation (ETI) in paediatric patients. The aim of the study was to compare the success rate of blind ETI through Blockbuster^TM^ laryngeal mask (LM) and air-Q® ILA in paediatric patients.

**Methods:**

Eighty patients with the American Society of Anesthesiologists’ physical status I and II, aged between six months and 10 years, were enrolled in this randomised controlled trial. The patients were intubated through either of the supraglottic airway devices (SADs) by visualised, blind intubation. The primary outcome was the first- attempt success rate of ETI. Secondary outcomes were the overall success rate of ETI, the oropharyngeal leak pressure, the fiberoptic glottic view, the time to intubation, and the complication rate.

**Results:**

Blockbuster^TM^ LM was having significantly higher first attempt success rate without any manipulation as compared to air-Q^®^ ILA (55% vs. 32.5%; *P* value =0.042). Overall success rate was also significantly higher in Blockbuster^TM^ LM (77.5% vs. 55%; *P* value =0.03). In subgroup analyses, Blockbuster^TM^ LM demonstrated significantly higher first-attempt and overall intubation success rates in children aged 6 months to 5 years, while success rates were comparable between devices among children older than 5 years.

**Conclusion:**

Blockbuster^TM^ LM, having more than 50% success rate of first-attempt blind intubation through SAD, can be a helpful device in crises during airway management of children and, thus, an ideal SAD for difficult airway carts.

**Main Points**

• Only two devices [air-Q® intubating laryngeal airway (ILA) and AmbuAura-i] have been studied for blind endotracheal intubation through supraglottic airway devices (SADs) in paediatric patients; air-Q® ILA has been found to have a better first attempt success rate among the two, ranging from 15-64%.

• Blockbuster^TM^ laryngeal mask (LM) is a newer SAD with a significantly better first attempt and overall success rate of blind intubation in children than air-Q® ILA.

• Blockbuster^TM^ LM having significantly higher oropharyngeal leak pressures than air-Q^®^ ILA, is better suited as a ventilatory device in surgeries requiring higher airway pressures.

## Introduction

Airway management in children can sometimes be particularly challenging and may require essential skills. The reported incidence of difficult intubation is 0.24-4.7% and 0.07-0.7% in infants and older children, respectively.^1^ As attempts at laryngoscopy increase in children, it is associated with high failure rates and incidence of severe respiratory complications responsible for perioperative morbidity and mortality.^2^ According to the paediatric perioperative cardiac arrest registry, 27% of all cardiac arrests occur due to respiratory-related events.^3^

The use of supraglottic airway devices (SADs) as a conduit to endotracheal intubation (ETI) (with or without flexible bronchoscopic guidance) is an accepted technique that has gained recognition on the emergency pathway of the American Society of Anesthesiologists (ASA) paediatric difficult airway algorithm.^4^ as well as in the Difficult Airway Society (DAS) guidelines.^5^

SADs can be used as a conduit for tracheal intubation, either with a fiberoptic bronchoscope (FOB) or blindly (without flexible bronchoscopic guidance). The success rate of ETI through these SADs ranges widely from 15% to 97%, depending on the type of SADs used, characteristics of patients, and operator-acquired skills.^6, 7, 8^

Based on various studies conducted in adults, the Fastrach laryngeal mask (LM) Airway or intubating LMA is recommended as a prototype standard reference device for blind ETI, owing to its great success^9^ but its paediatric size is not available.^10^ Only two devices [air-Q® intubating laryngeal airway (ILA) and AmbuAura-i] have been studied for blind ETI through SADs in paediatric patients; air-Q® ILA has been found to have a better first-attempt success rate between the two, ranging from 15-63%.^11, 12, 13, 14, 15, 16^

Blockbuster^TM ^LM has a success rate of 90% for blind ETI in adults.^8^ Limited paediatric data are available, with recent evidence evaluating Blockbuster^TM ^LM primarily as a conduit for fiberoptic-guided intubation in children.^17, 18^ However, its performance for blind intubation, which may be required in emergency or resource-limited settings, remains inadequately studied. The proposed study aims to answer the research question that which SAD (Blockbuster^TM ^LM or air-Q®ILA) has a higher first-attempt success rate for blind ETI in paediatric patients. The results can be further extrapolated to the use of SAD when FOBs are limited.

The study’s primary outcome was the first-attempt success rate of ETI. Secondary outcomes were the overall success rate of ETI, the number of attempts required for successful placement of SAD, the oropharyngeal leak pressure (OLP), the glottic view through a FOB, the time needed for successful intubation, and the incidence of peri- and post-procedural complications.

## Methods

This single-center, parallel-group, randomized controlled trial received ethical approval from the All India Institute of Medical Sciences, Patna ethics committee (approval: AIIMS/Pat/IEC/PGTh, date: 25.03.2021). It was then conducted in a tertiary healthcare setting and registered in the Clinical Trials Registry of India (CTRI/ 2021/ 03/ 032076). Recruitment was performed between March and October 2021. Written informed consent was obtained from the patient’s legal guardians, and oral assent was taken from the child wherever applicable. This manuscript adheres to the applicable CONSORT guidelines.

Paediatric patients with ASA-physical status (ASA-PS) I and II aged between 6 months and 10 years, weighing 5 to 30 kg, and scheduled for elective surgery under general anaesthesia were recruited for the study. Patients with an anticipated difficult airway, significant cardiopulmonary disease, obesity, airway surgery, or expected postoperative admission to the intensive care unit were excluded from the study. 104 patients were assessed for eligibility, and 80 were randomised into two equal groups.

A block randomisation sequence list was created online (https://www.sealedenvelope.com) by an individual not involved in the study. The opaque envelope method of concealment was used in the study. After enrollment, envelopes were opened by an independent person on the morning of surgery, and patients were allocated to two groups according to the sequence number in a 1:1 ratio.

### Group 1: Airway secured with air-Q®ILA

### Group 2: Airway secured with Blockbuster^TM ^LM

Each group consisted of 40 patients. All the children were kept nil by mouth according to standard guidelines. No premedication was administered to the patient.

Inhalational induction with sevoflurane was performed, followed by administration of fentanyl at 2 µg kg^-1^. The neuromuscular blockade was achieved with atracurium at 0.5 mg kg^-1 ^after confirming mask ventilation. After ventilating the patient for 3 minutes, achieving a minimum alveolar concentration of 1.0-1.2, the airway was secured with an appropriately sized SAD according to the patient’s weight per the allotted group. The air-Q® ILA sizes 1, 1.5 and 2 were used for the patient weight of (<7 kg), (7-17) kg and (17-30) kg, respectively. The Blockbuster^TM ^LM sizes of 1.5, 2, and 2.5 were used for patient weights of (5-10) kg, (10-20) kg, and (20-30) kg, respectively.

All airway interventions were performed by the investigators, who were certified and trained anaesthesiologists. The performers had experience using other SADs (more than 100) in children as part of routine practice. The performers were trained on manikins with the SADs used in the study and used them on a limited number of patients before the study.

The SADs were lubricated with a water-soluble agent and inserted using the recommended midline technique. A maximum of three SAD insertion attempts with minor airway interventionswas allowed. Successful placement of SAD was determined by achieving at least 5 ml kg^-1 ^tidal volume, bilateral chest excursion, and a square-wave capnogram upon delivery of a positive-pressure breath, with a tidal-volume leak <10%. The OLP was measured by closing the expiratory valve of the circle system with a fresh gas flow of 3 L min^-1^ and noting the airway pressure at which equilibrium was reached. The pressure was not allowed to exceed 40 cm of H_2_O.

**Figure 1:**
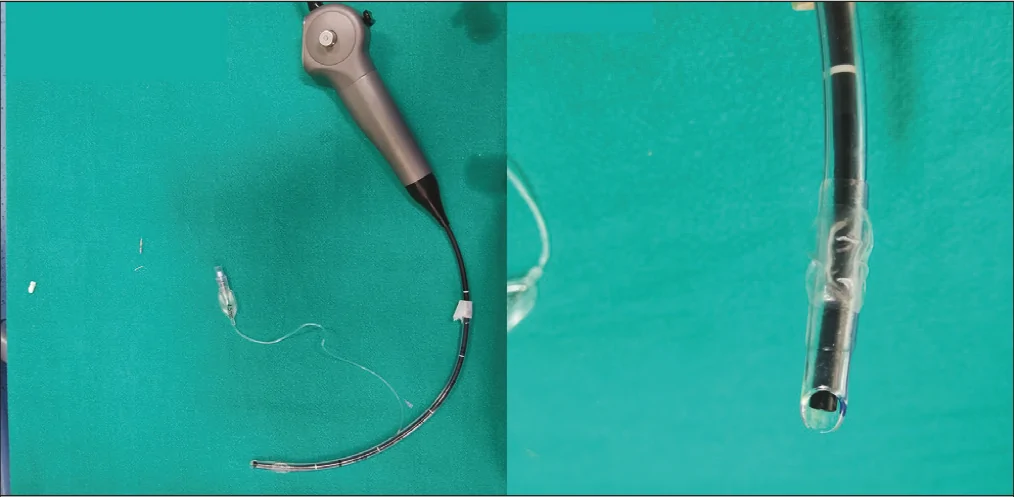
ETT fixation over the FOB for visualised blind intubation. ETT, endotracheal tube; FOB, fiberoptic bronchoscope.

**Figure 2:**
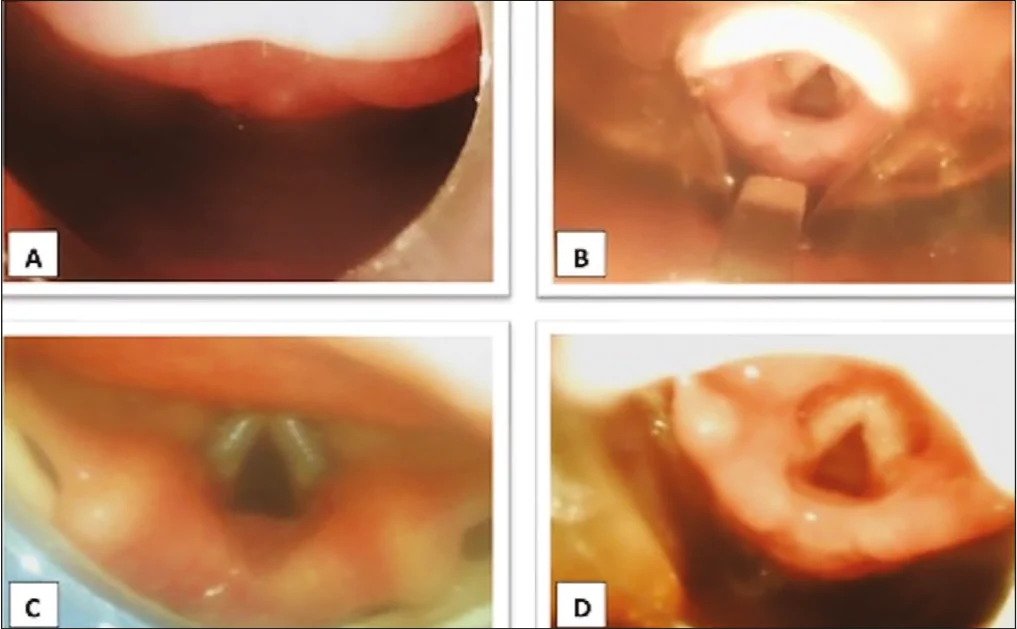
Brimacombe’s grade for fibreoptic glottic view. A: Grade I; B: Grade II; C: Grade III; D: Grade IV.

Volume-controlled ventilation mode was used with a target tidal volume of 8-10 mL kg^-1 ^and a respiratory rate of 14-20 times per minute to maintain an EtCO_2_ between 30-40 mm of Hg. A paediatric FOB (Karl Storz, Tuttlingen, Germany) was fitted with a cuffed polyvinyl chloride endotracheal tube (ETT) of a size appropriate for the patient’s age. The ETT was taped to the FOB so that the tip of the FOB did not extend beyond the ETT (Figure 1). The breathing circuit was disconnected at the patient end, and the ETT-loaded FOB was inserted through the SAD. During the visualisation of the laryngeal structures, a fiberoptic view through the opening of the SAD was recorded by a member of the anaesthesia care team. Scoring of the fibreoptic glottic view was based on Brimacombe’s laryngeal view and was recorded as follows (Figure 2):^19^

1. Grade 0: failure to function of LMA where cords are not seen fibre-optically,

2. Grade 1: cords not seen, but the function of LMA is adequate,

3. Grade 2: The cords and the anterior epiglottis are visible,

4. Grade 3: cords plus posterior epiglottis are seen,

5. Grade 4: only cords are seen.

For glottic view grades 2, 3, and 4, we attempted ETI. We used a visualised blind technique for intubation, as previously described in the literature, to prevent injury to the delicate laryngeal structures of the child.^11, 20, 21^ To reduce performer bias, the performer did not view the fiberoptic screen during intubation. The FOB did not guide the insertion of the ETT but, instead, followed its progress (Figure 1). The performer was also not allowed to use the fiberscope’s lever. An assisting anaesthesiologist communicated with the performer regarding the advancement of the ETT by using only the terms “Go,” “Slow,” “Stop,” and “Back” after the visualisation of the FOB laryngeal-view grade.

The first successful attempt at ETI was defined when ETT was advanced directly into the glottic opening and confirmed by capnography. If the ETT deviated into extraglottic structures during the procedure, corrective manoeuvres, such as mandibular lift and rotation of the ETT, were used. If the grade improved, we proceeded with ETI; if the grade did not improve, it was considered a failed attempt at blind intubation. ETI, with corrective manoeuvres, was considered a subsequent attempt at intubation. Correction manoeuvres were also applied to the grade 1 glottic view.

Patients were ventilated for 3 minutes after successful ETI, after which the SAD was removed using the removal stylet. One visualised blind intubation attempt lasting no more than 2 minutes was allowed. The intubation was recorded as a failure:

1. The correct placement of the SADs was not achieved after three attempts despite minor airway interventions.

2. The tracheal tube was dislodged during SAD removal.

3. Deviation of ETT away from the laryngeal inlet despite using correction manoeuvres.

The various endpoints were defined as follows:

1. Time for the successful placement of SAD: From discontinuation of facemask ventilation to the rise of 1st square wave of the capnographic waveform.

2. Time for the successful insertion of the ETT: From discontinuation of the breathing circuit after SADs placement to 1st square wave of the capnographic waveform.

3. Time for the successful removal of SAD: From disconnection of the breathing circuit after successful ETI till reconnection of the breathing circuit to the ETT.

All outcome variables were noted in the data collection proforma by a member of the anaesthesia care team. Adverse events such as aspiration or regurgitation, hypoxia, bronchospasm, laryngospasm, desaturation (SpO_2_<92%), coughing, blood-staining on the SAD, and trauma to teeth, tongue, or lips were recorded. Patients older than three years were observed for sore throat, postoperative nausea and vomiting, hoarseness, and dysphagia in the post-anaesthesia care unit. A follow-up twenty-four hours after surgery and interviews about side effects were conducted with the child and the parents.

### Statistical Analysis

The authors conducted a pilot study on 12 patients in each group, the success rates of Blockbuster^TM ^LM and air-Q® being 66% and 33%, respectively. The calculated sample size was 35 in each arm, considering a power of 80%, a 95% confidence interval, and a two-tailed alpha of 0.05. We planned to recruit 40 patients in each arm, allowing for dropouts.

All data were recorded on a standardised data collection sheet, and were analysed using the statistical software SPSS 26.0 (SPSS, Chicago, IL, USA). The normality of the data was tested using visual inspection of the Q-Q plot and the Shapiro-Wilk test. Descriptive parametric data were expressed as mean ± standard deviation and non-parametric data as median and interquartile range. Parametric continuous variables were compared using the independent-samples t-test. Non-parametric continuous variables were compared using the independent-samples Mann-Whitney U test. Categorical and ordinal data were compared using the chi-square test or Fisher’s exact test. A post-hoc, age-based subgroup analysis was performed for outcomes including the first-attempt and overall success rates of ETI. A *P* value of < 0.05 was considered statistically significant.

## Results

**Figure 3:**
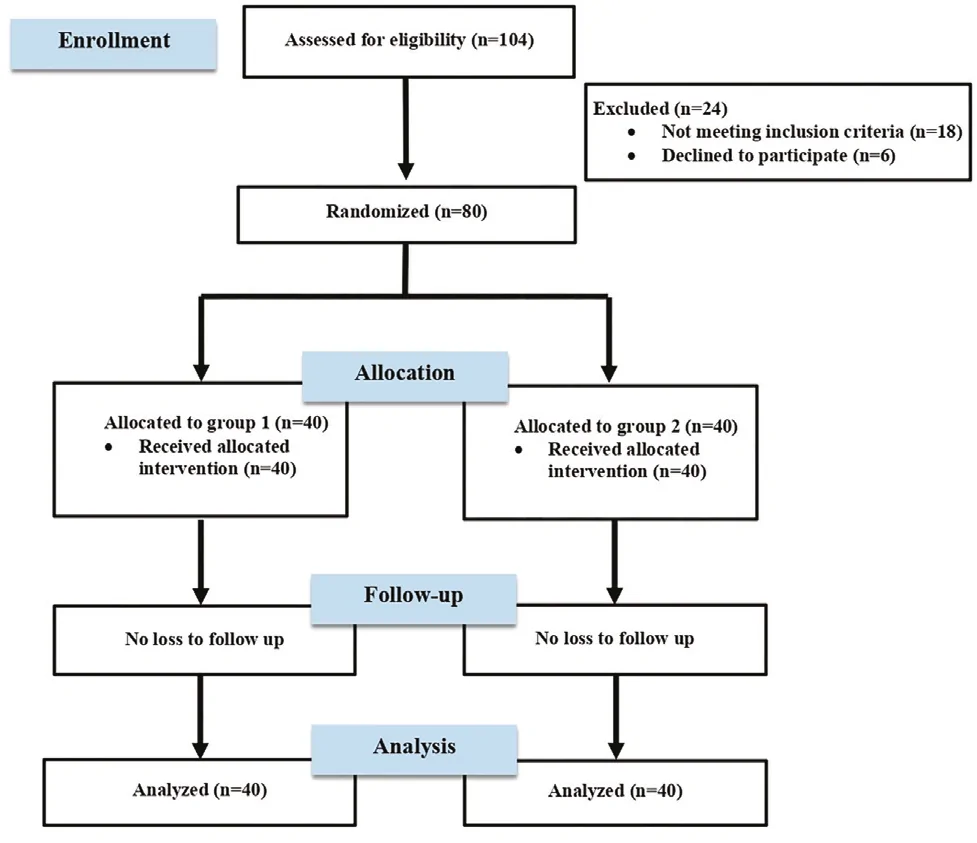
CONSORT flow diagram.

Of the 104 patients assessed for eligibility, 18 did not meet the inclusion criteria, and consent for participation in the study could not be obtained from the guardians of 6 patients. The remaining 80 patients were randomised to two study groups and underwent interventions per protocol, as represented in the CONSORT flow diagram (Figure 3).

Groups 1 and 2 each had 40 patients at the final analysis. There were no statistically significant differences between the groups regarding demographic data (age, gender, height, weight, body mass index, ASA-PS) (Table 1).

Group 2 had a significantly higher first-attempt success rate than Group 1 (55% vs. 32.5%; *P* value =0.042) (Table 2). The overall success rate of ETI was significantly higher in Group 2 than in Group 1 (77.5% vs. 55%; *P* value =0.03) (Table 2).

In the subgroup of children aged 6 months to 5 years (n = 57), the first-attempt success rate for ETI was significantly higher in Group 2 than in Group 1 (53.8% vs. 25.8%; *P*=0.030). The overall success rate was also significantly higher in Group 2 (73% vs. 45.2%; *P*=0.033). In children aged >5-10 years (n = 23), no statistically significant difference was observed between the two devices for either first-attempt success (57.1% vs. 55.5%; *P*=0.94) or for overall success rate (85.7% vs. 77.7%; *P*=0.624) (Table 3).

SAD was placed in a single attempt in all patients except one in Group 2, in whom placement was unsuccessful despite the maximum allowable attempts (three, per protocol). Failure to place the SAD was counted as an unsuccessful intubation attempt.

The OLP (in cm of H_2_O) was significantly higher in Group 2 than the OLP of Group 1 (25.74±7.05 vs. 15.2±5.58; value =0.001) (Table 2).

FOB visualisation of vocal cords (Grades 2/3/4) was observed in 62.5% of Group 1 patients versus 84.6% of Group 2 patients (*P* value =0.026). This variation was statistically significant (Table 2).

The time (in seconds) required for successful insertion of ETT was significantly less in Group 2 [60 (53-60)] than in Group 1 [50 (39-60)] (*P* value =0.019) (Table 2).

The time required for SAD insertion, removal, and tidal volume leak was not statistically significant (Table 2).

The SAD sizes used in both groups are presented in Table 2. The statistical analysis of the sizes cannot be interpreted because the weight corresponding to specific sizes differed between the two groups , in accordance with the manufacturer’s recommendations.

The number of patients successfully intubated after using corrective manoeuvres was 8 in Group 1 and 9 in Group 2 (Table 2).

There was no significant difference in the incidence of complications between the two groups (Table 4).

## Discussion

In the present study, both the first attempt and overall success rate with Blockbuster^TM^ LM were significantly better compared to air-Q® ILA (55% and 77.5% vs. 32.5% and 55%). The age-based subgroup analysis provides important insights into how paediatric airway development influences intubation success. Among children aged 6 months to 5 years, Blockbuster^TM^ LM demonstrated significantly higher first-attempt and overall success rates than air-Q® ILA. This finding is clinically relevant because younger children have a relatively larger, floppier epiglottis, a higher, more anterior larynx, and reduced alignment of the SAD with the glottic opening. These anatomical features may favour devices with a guided tube-directing mechanism, such as the Blockbuster^TM^ LM. In contrast, children older than 5 years have airway anatomy that is more adult-like, with improved laryngeal alignment and increased epiglottic rigidity, which likely explains the comparable success rates observed between the two devices in this age group.

In a previous study in adults, Blockbuster^TM^ LM had a first-attempt blind ETI success rate of 90%.^8^ The success rate of blind ETI through air-Q® ILA in paediatric patients is 15-63% in various studies.^11, 12, 13, 14, 15, 16^ and is the highest among all the SADs that have been studied in paediatric patients. Soni et al.^17^ and Chauhan et al.^18^ evaluated Blockbuster^TM^ LM and air-Q® ILA as conduits for fiberoptic-guided intubation in children and reported comparable intubation success rates between the devices. In contrast, the present study focuses on visualised-blind intubation which is specifically relevant to emergency, resource-limited, and crisis airway scenarios, and additionally reports OLPs and a subgroup analysis of younger children.

Blockbuster^TM^ LM is a newer SAD with unique features like a gastric inlet port and a guidance device that allows the ETT to be directed towards the laryngeal opening at 30-degree angle. These features are expected to increase the success rate of ETI.

As defined by TM Cook, the suffix “i” should be added to those devices that enable intubation (e.g., with success >50%),^22^ and thus, these SADs can be helpful in situations where the use of FOBs is limited. Blockbuster^TM^ LM, having a better success rate of blind intubation, can be a preferred device for managing crises during difficult airway management of children.

OLP is considered a good measure of airway protection, of successful SAD placement with a better seal, and of adequate positive pressure ventilation. In the present study, the OLP in Blockbuster^TM^ LM was significantly higher than air-Q® ILA [25.9 (22-32) vs. 12 (11-20.2)]. Both SADs have an adequate OLP, with a tidal-volume leak of only 1-3%. W e suggest that both SADs can be used effectively as ventilatory devices. SADs are successfully being used as a primary ventilatory device in paediatric patients undergoing laparoscopic surgeries.^23^ Blockbuster^TM^ LM, due to better OLP, can be used as an effective device for surgeries requiring higher ventilatory pressures, like laparoscopic surgeries, obese patients, and Trendelenburg position. A recent study has also shown better OLP of BlockbusterTM LM (24.72±6.81 cm H_2_O), similar to our findings.^24^

Various manoeuvres have been described to facilitate insertion and ETI via SADs, such as mandibular lift, ETT rotation, and the Chandy manoeuvre. Intubation was successful in about 20% of patients in both groups after corrective manoeuvres were applied. Manoeuvres were not helpful for any patients with Grade I glottic views. Manoeuvres were unsuccessful in 70% and 47% with air-Q® ILA and Blockbuster^TM^ LM, respectively. No injuries or complications occurred during the attempt at corrective manoeuvres. We suggest that correction manoeuvres should be applied on subsequent attempts after encountering resistance during the advancement of the ETT or after a failed first attempt.

There is a potential chance of airway trauma and epiglottic down folding during blind attempts of ETI in children.^25, 26^ Therefore, to avoid trauma to the delicate paediatric airway, we used a “visualised blind intubation” technique. The number of patients with an unsuitable glottic view (grade 1) was significantly higher in the air-Q® ILA group than in the Blockbuster^TM^ LM group (37.5 % vs. 15.4 %). In the Blockbuster^TM^ LM group, although the optimal glottic view was achieved in 84.6% of children, the intubation success rate was lower than expected. This might be due to the smaller size of the glottic opening in children compared to adults. Moreover, the Parker Flex-Tip tracheal tube is recommended by the manufacturers of Blockbuster^TM^ LM for blind tracheal intubation. This use of the tube might have increased the success rate further.

SAD insertion was successful in the first attempt in all patients except one with Blockbuster^TM^ LM. Meta-analysis on the use of air-Q® ILA in paediatric patients has also shown similar results, with a 100% success rate of device insertion in most of the studies.^13^ Blockbuster^TM^ LM in adult patients also had a 100% first-attempt success rate.^8^ SAD insertion and removal times were similar between the two groups. ETT insertion time was significantly longer with air-Q® ILA compared to Blockbuster^TM^ LM (60 vs. 50 seconds). However, this time difference of 10 seconds is clinically insignificant in routine practice.

Complications during ETI through SADs were neither statistically nor clinically significant.

### Study Limitations

Our study has limitations that should be considered when interpreting our results. First, we have not included patients younger than six months of age in our research. The infants have large, floppy epiglottises, which prevent the visualisation of the glottis. Thus, the success rate in infants may be lower than in older children. Second, we selected patients with body weights ranging from 5 kg to 30 kg; therefore, the findings might not apply to underweight or overweight patients. Third, the subgroup analysis was post hoc and not powered a priori; therefore, these findings should be interpreted cautiously. Fourth, we have used a visualised-blind method of ETI, rather than truly blind ETI, to avoid airway injury. The visualised blind technique prevented us from advancing the ETT in the event that it deviated from the correct path. The true blind technique might have resulted in inadvertent, forceful insertion, which, at the expense of causing airway trauma, might have increased the first-attempt success rate. Fifth, because our study included patients with normal airways, the results may not apply to patients with difficult airways. We suggest further research comparing SADs as intubating devices in infants.

In general, it is advisable to use fiberoptic-guided intubation through an SAD. However, in some situations, fiberoptic-guided intubation may be difficult, such as when bleeding or secretions are present. In prehospital settings or by novice physicians, blind intubation through SAD may be attempted when a definitive airway is required during an emergency. Blind ETI through SAD is also helpful during airway crises in children.

## Conclusion

Blockbuster^TM^ LM, having a 50% to 77.5% success rate of blind intubation through SAD, can be a helpful device in crises and, thus, a preferred SAD for difficult airway carts. Both air-Q® ILA and Blockbuster LM have a first-attempt SAD insertion success rate of about 100% with non-significant tidal volume leak. Therefore, both devices can be used effectively for ventilation. Blockbuster^TM^ LM has a significantly higher OLP than air-Q® ILA; therefore, BlockbusterTM LM can be used as a better ventilatory device in surgeries requiring higher airway pressure, like laparoscopic surgeries or obese patients.

## Ethics

**Ethics Committee Approval:** This single-center, parallel-group, randomized controlled trial received ethical approval from the All India Institute of Medical Sciences, Patna ethics committee (approval: AIIMS/Pat/IEC/PGTh, date: 25.03.2021).

**Informed Consent:** Written informed consent was obtained from guardians.

## Figures and Tables

**Table 1. Demographic Data:** 

**Characteristics**	**Group 1, n = 40**	**Group 2, n = 40**	***P*** **value**
**Age** **(months)**	46 (37-53)	54 (46-62)	0.138‡
**Weight** **(kg)**	14.27 (12.39-16.44)	15.78(14.08-17.47)	0.117‡
**Height** **(cm)**	98.7 (94.1-103.4)	103.4 (98.3-108.5)	0.174‡
**BMI**	14.13 (13.28-14.98)	14.51(13.59-15.42)	0.544‡
**Sex** **(M/F)**	24/16	28/12	0.482^†^
**ASA-PS** **(I/II)**	38/2	36/4	0.675^¶^
**SAD** **size** **(1.5/2/2.5)**	22/18	3/27/10	-

Values are presented as median (IQR) and frequency. *P* < 0.05 =Significant; ^†^:chi square test, ‡: Mann whitney U test, ^¶^:Fisher exact test

BMI, body mass index; ASA-PS, American society of Anesthesiologists physical status; SAD, supraglottic airway device; IQR, interquartile range

**Table 2. Device Characteristics:** 

**Outcomes**	**Group 1, n = 40**	**Group , n = 40**	**Relative risk, (95% CI)**		***P* value**
***Primary*** ***outcome***					
First attempt successful ETI	13/40 (32.5%)	22/40 (55%)		0.66 (0.44 to 0.99)	0.042^†^
***Categorical*** ***secondary*** ***outcomes***					
Overall success rate of ETI	22/40 (55%)	31/40 (77.5%)		0.67 (0.48 to 0.95)	0.03^†^
Successful SAD insertion	40/40 (100%)	39/40 (97.5%)		-	-
Good glottic visualisation (Grade 2-4)	25/40 (62.5%)	33/39 (84.6%)		0.73 (0.56 to 0.97)	0.026^†^
Successful ETI with maneuvers	8/40 (20%)	9/39 (23%)		0.86 (0.37 to 2.01)	0.785^†^
First attempt success with SAD sizes (1.5/2/2.5)	18%/50%/ -	67%/52%/60%		-	-
***Continuous*** ***secondary*** ***outcomes***				**Difference** **in** **means** **(95%** **CI)**	-
Time for SAD insertion (seconds)	24 ±7 (n=40)	23±9 (n = 39)		0.77 (-2.84 to 4.38)	0.672^§^
OLP (cm of H_2_O)	15.2±5.58 (n = 40)	25.74±7.05 (n = 39)		-10.5 (-13.4 to -7.7)	0.001^§^
-				**Difference** **in** **medians** **(95%** **CI)**	-
Time for SAD removal (seconds)	27 (22-32) n = 21	24 (21-36) n = 31		2 (-3 to 6)	0.484‡
Time for successful ETI (seconds)	60 (53-60) n = 21	50 (39-60) n = 31		5 (0 to 11)	0.019‡
Tidal volume leak in percentage	4 (0-6) n = 40	0 (0-0) n = 39		-	0.118‡

Values are presented as mean ± SD; median (IQR). *P* < 0.05 = Significant; ^†^:chi square test, ^§^: Independent sample t-test, ‡: Mann-whitney U test

CI, Confidence interval; OLP, oropharyngeal leak pressure; FOB, fiberoptic bronchoscopy; ETI, endotracheal intubation; SAD, supraglottic airway device; IQR, interquartile range; SD, standard deviation

**Table 3. Subgroup Analysis of Successful Attempts:** 

**Outcomes**	**Group 1, n = 40**		**Group 2, n = 40**	**Relative risk, (95% CI)**	***P* value**
**Age** **6** **months** **to** **5** **years** **(n** **=** **57)**					
First attempt successful ETI		8/31 (25.8%)	14/26 (53.8%)	1.81 (0.98 to 3.30)	0.030^†^
Overall Success rate of ETI		14/31 (45.2%)	19/26 (73%)	1.67 (1.04 to 2.68)	0.033^†^
**Age** **>5** **years** **to** **10** **years** **(n** **=** **23)**					
First attempt successful ETI		5/9 (55.5%)	8/14 (57.1%)	1.04 (0.37 to 2.90)	0.940^†^
Overall success rate of ETI		7/9 (77.7%)	12/14 (85.7%)	1.36 (0.43 to 4.26)	0.624^†^

Values are presented as frequency (percentage). *P* < 0.05 = Significant; ^†^:chi square test

CI, confidence interval; ETI, endotracheal intubation

**Table 4. Complications During Procedure:** 

**-**	**Group 1, n = 40**	**Group 2, n = 40**	***P* value**
During SAD insertion	0	0	-
During removal of SAD (blood tinge at tip)	0, n = 40	1, n = 39	0.494^¶^
During extubation (laryngospasm/excessive cough)	4, n = 21	4, n = 31	1.000^¶^
Desaturation (SpO_2_<90%)	0	0	-
Sore throat	6	5	0.745^†^
Hoarseness of voice	7	6	0.762^†^

Values are represented in frequency. Frequency was compared using a chi-square test ^†^ or ^¶^: Fisher exact test

n, number of patients; SAD, supraglottic airway device; SpO_2_, oxygen saturation
